# Podiatry care in rheumatoid arthritis: differences between current and ideal service provision

**DOI:** 10.1186/1757-1146-6-S1-O16

**Published:** 2013-05-31

**Authors:** Kym Hennessy, Anita Williams, Martijn Steultjens, James Woodburn

**Affiliations:** 1 Institute for Applied Health Research, School of Health & Life Sciences, Glasgow Caledonian University, UK; 2 Department of Podiatric Medicine, School of Science & Health, University of Western Sydney, Australia; 3 Directorate of Prosthetics, Orthotics and Podiatry, School of Health Science, University of Salford, UK

## Background

Foot and ankle involvement in rheumatoid arthritis (RA) is common, impacting negatively on quality of life. Stakeholder perceptions of podiatric service provision are unknown. Given the importance of specialist podiatry care, this knowledge would be beneficial. This study explored opinions of people with RA and podiatrists regarding current and ideal podiatry care in NHS Scotland.

## Methods

Two focus groups were conducted with participants from five NHS Health Boards in Scotland. One focus group involved people with RA who previously received podiatry care, and the other, podiatrists who treat people with RA. The Framework approach was used to identify core concepts and associated themes.

## Results

Five people with RA (all female) with mean ± SD age of 53.6 ± 6.6 years and disease duration 15 ± 11 years participated in the first focus group. The average duration of podiatry care was 7 years (range 3-15). Six rheumatology specialist podiatrists participated in the second focus group. Both groups identified similar issues with current care and steps that could be taken to achieve ideal service provision. ‘Access to health care services’ (core concept one) had associated themes of ‘access facilitated’ and ‘access inhibited’. ‘Tailored podiatry service for the complex needs of people with RA’ (core concept two) had associated themes of ‘podiatry service location’, ‘profile of podiatry’, ‘foot health interventions’, ‘podiatrist skills’, and ‘service review’. ‘Tailored service’ also emerged from the podiatrist focus group.

## Conclusions

Podiatry care was regarded as a positive and important part of overall care for people with RA. However, more integrated specialist services with moves towards a national model of care may be beneficial. Participating podiatrists widely endorsed these themes. Greater concurrence between stakeholders could lead to more flexible and accessible services better meeting patient need.

